# Phytochemical Analysis and Characterisation of Brewers’ Spent Grain Properties as Affected by Fermentation and Ultrasonication Pretreatments

**DOI:** 10.3390/foods14203579

**Published:** 2025-10-21

**Authors:** Sogo James Olatunde, Tumisi Beiri Jeremiah Molelekoa, Oluwafemi Ayodeji Adebo

**Affiliations:** 1 Centre for Innovative Food Research (CIFR), Department of Biotechnology and Food Technology, Faculty of Science, University of Johannesburg, Doornfontein, P.O. Box 17011, Johannesburg 2094, South Africa; sjolatunde@lautech.edu.ng; 2 Department of Food Science, Ladoke Akintola University of Technology, Ogbomoso P.M.B. 4000, Oyo State, Nigeria

**Keywords:** BSG, fermentation, thermal properties, ultrasonication, antioxidant activity

## Abstract

Brewers’ spent grain (BSG) is a highly abundant, nutrient-rich by-product generated by the brewing sector. Upcycling and reusing by-products from the food sector have become necessary to achieve sustainable food security globally. This research investigated traditional and novel pretreatments to modify the properties of BSG for better utilisation as a food ingredient. In this study, BSG was ground and subsequently processed in two different ways: ultrasonicated for 15 min and fermented with lactic acid bacteria for 24, 48, and 72 h. Influences of fermentation and ultrasonication on the antioxidant activity, thermal properties, colour, chemical composition, total phenolic content (TPC), and total flavonoid content (TFC) were then investigated. There was a general increase in the antioxidant properties of BSG flour ultrasonicated for 15 min (FRAP, 4.35 mgTE/g; DPPH, 65.87%; ABTS, 37.29%) and fermented BSG flour fermented with *Lactococcus lactis* (48 h) (FRAP, 2.29 mgTE/g; 63.93%; 24.48%) compared to native BSG (2.10 mgTE/g, FRAP; 65.87%, DPPH; 23.50%, ABTS). The highest percentage of fibre (28%) was observed in BSG fermented for 24 h. There was also increase in colour value (L*, 56.33–59.07; a*, 3.18–3.65; b*, 9.61–10.70), TPC, and TFC (0.13–0.16 and 0.37–1.30, respectively), and variations in peak intensities on the thermogram. These results indicate that ultrasound and fermentation are promising technologies for the enhanced valorisation of BSG for value-added food product development.

## 1. Introduction

Globally, there is considerable pressure to minimise the volume of waste generated by industries [1]. The food industry produces high volumes of by-products with valuable compositions that could still find application as starting materials for further processing. This has led researchers to explore different possibilities for utilising various by-products to produce novel products [2].

Brewers’ spent grain (BSG) is the main by-product of the brewing sector, comprising the outer layers of the barley kernels and other cereals containing fibrous residue generated during the brewing process [3]. Large quantities of BSG are produced during the wort production stage, with an estimate of about 85% of by-products from about 40 million tonnes of wort produced globally [4,5]. Of the total BSG produced globally, about 70% is used for animal feed, 10% for biogas generation, and 20% is discarded in landfills or for low-value applications [6].

Despite its relatively low applications, BSG has become an attractive material with the potential for use as value-added products (VAPs) due to its low cost [7], high availability, and rich nutritional quality. Therefore, there is a need for an innovative approach to utilising such agricultural waste rich in bioactive substances, to address the world’s food needs [8]. The proximate constituents of BSG include appreciable levels of dietary fibre, protein, fat, cellulose, hemicellulose, lignin, and essential amino acids with significant polyphenols, lipids, vitamins, and minerals [9]. The vitamins identified in BSG include riboflavin, thiamine, biotin, folic acid, niacin, choline, pyroxidine, and pantothenic acid, while the minerals present are calcium, copper, iron, manganese, potassium, and sodium [10,11,12]. It is reportedly a valuable source of phenolic acids, particularly ferulic and p-coumaric acids [13].

Various aspects of BSG chemical constituents have recently been studied, including the preservation of its bioactive components [14,15]. Similarly, the antioxidant, functional, thermal and rheological properties of BSG have been studied, as well as the effect of the ultrasonication pretreatment of BSG for maximum recovery of cellulose and hemicellulose for bioethanol production [16]. A complete characterisation of BSG to determine its physical, chemical, thermal, morphological, structural, and surface properties has also been reported [17]. However, variations noted could owe to differences in barley type, harvesting period, properties of the hops used, and the brewery process.

Different studies have reported the benefits of fermentation and ultrasonication on the overall composition of food [18,19]. Considering the need to further improve the value of BSG prior to utilisation in product development, this study investigated the beneficial influence of fermentation and ultrasonication pretreatments on BSG, and characterised the colour, total phenolic and flavonoid contents, antioxidant activity, and chemical and thermal properties.

## 2. Materials and Methods

### 2.1. Sample Collection and Preparation

A brewery in Roslyn, Pretoria, South Africa, generously supplied dried BSG. The dried BSG was ground (platinum dry miller KJ-1250, Castelfranco Veneto, Italy), sieved using a 500 mm sieve, and kept at ambient temperature in polyethylene bags for further analysis. Lactic acid bacteria (LAB) starter cultures YC-XII and CHN-22, stored at −18 °C, were purchased from Horsholm Denmark. All other reagents utilised in this study were of analytical grade.

### 2.2. Sample Pretreatments

#### 2.2.1. Fermentation

BSG native flour of 100 g was mixed with 0.4 g lactic acid starter culture (YC-XII or CHN-22) in a container filled with 100 mL sterile distilled water. The mixture was fermented for 24 h, 48 h, and 72 h at 35 °C, respectively, in an incubator (Incotherm, Labotec, Johannesburg, South Africa). After the fermentation period, the samples were freeze-dried using a freeze drier (Harvest plus freeze dryer, Salt Lake City, UT, USA) at 18 °C for 72 h, after which the biomass was milled and subsequently sieved through a 500 µm mesh (Analysette 3 Spartan, Fritsch GmbH, Idar-Oberstein, Germany) and kept at room temperature (25–30 °C) for subsequent analysis [20]. An uninoculated native BSG sample was used as a control.

#### 2.2.2. Ultrasonication

The method of Cui and Zhu [21] was adopted with slight modifications, to produce ultrasonicated flour samples using a Misonix Ultrasonic Liquid processor (Fisher Scientific TI-H-10 Ultrasonic bath, output 750 W, Waltham, MA, USA). About 30 g of BSG flour was mixed with 100 mL of distilled water in a 150 mL beaker and held in an ultrasonic water bath for 15 min and 30 min, respectively. The mixture was then transferred into a freezer. The frozen samples were freeze-dried (Harvest plus freeze dryer, Salt Lake City, UT, USA) at 18 °C for 72 h and then milled into flour and, subsequently, sieved through a 500 µm mesh (Analysette 3 Spartan, Fritsch GmbH, Idar-Oberstein, Germany), packaged in a Ziploc^®^ bag (Shoprite Holdings Ltd., Cape Town, South Africa), and kept at room temperature (25 °C) for subsequent analysis.

### 2.3. pH and Titratable Acidity

The pH of the native BSG and the pretreated BSG samples was analysed using a standardised pH metre (EcoSense pH10A, YSI Inc., Taus, Singapore), whereby 10 g of the sample was mixed with 90 mL distilled water. Titratable acidity (TTA) was determined by titrating the aliquot with NaOH (0.1 M) until it reached a pH of 8.5. TTA results were recorded as the total NaOH volume (mL) used [22].

### 2.4. Colour Measurement

The colour values, L*, a*, and b*, of the BSG samples were determined using a chroma metre, whereby L* is lightness, a* is the degree of redness or greenness, and b* is yellowness or blueness. Samples were placed on a transparent glass plate along with the chroma metre and determined in triplicate. The chroma metre (Minolta Chroma Meter CR-400, Minolta Co. Ltd., Osaka, Japan) was calibrated using a whiteboard [23].

### 2.5. Proximate Analysis of BSG Flour

The percentages of moisture content, protein, crude fibre, fat, and carbohydrates in the BSG flour were determined using the protocol by the AACC [24].

### 2.6. Total Phenolic and Flavonoid Contents and Antioxidant Activity

#### 2.6.1. Methanolic BSG Flour Extraction

Briefly, BSG flour (0.25 g) was mixed with 80% aqueous methanol containing 1% HCl and the mix was subsequently sonicated using a water bath ultrasonicator (Argolab AU-220, Carpi, Italy) for one hour. The recovered extract tubes were centrifuged (10 min, 4 °C, 4000× *g*; Eppendorf 5702R, Hamburg, Germany) to obtain a supernatant and then filtered for subsequent analysis.

#### 2.6.2. Total Phenolic Content (TPC) of BSG

Ten microlitres of BSG extract was added to the reaction mixture (prepared Folin–Ciocalteu phenol reagent (50 µL), 7.5% Na_2_CO_3_) in a 96-well plate in triplicate. The plate incubation was conducted for 30 min and read at 750 nm using a microplate smartReader™ 96 (MR-9600, Accuris Instruments, Benchmark Scientific Inc., Edison, NJ, USA). The TPC was calculated as milligram gallic acid equivalent (mg GAE) per gramme of sample with gallic acid concentration as a standard.

#### 2.6.3. Total Flavonoid Content (TFC) of BSG

For the flavonoid extraction, BSG flour (0.25 g) was mixed with 80% aqueous methanol containing 1% HCl, and the mix was subsequently sonicated using a water bath ultrasonicator (Argolab AU-220, Carpi, Italy) for 1 h. The recovered extract tubes were centrifuged (10 min, 4 °C, 4000× *g*; Eppendorf 5702R, Hamburg, Germany) to obtain a supernatant and then filtered for subsequent analysis. Thereafter, 10 µL BSG extract was pipetted onto the reaction mixture (2.5% NaNO_2_, 1.25% AlCl_3_, 2% NaOH, and 10 µL extract) and quercetin concentrations (standard) were separately contained in a 96-well plate, and then the samples’ absorbance was read at 450 nm using a microplate smartReader™ 96 (MR-9600, Accuris Instruments, Benchmark Scientific Inc., Edison, NJ, USA). The TFC was recorded as milligramme quercetin equivalent (mg QE) per gramme of sample.

#### 2.6.4. Estimation of Antioxidant Efficacy (DPPH, ABTS, and FRAP Assays)

##### 2,2-Diphenyl-1-Picrylhydrazyl (DPPH)

The DPPH radical scavenging activity of the BSG extracts was kept at −20 °C in covered amber vials until their analysis. The absorbance was measured at 517 nm. The DPPH radical scavenging activity of free phenolics in the BSG extract solutions (10 mg/mL) samples and standard (Trolox) was measured. The DPPH values were expressed as mg of Trolox equivalents per gramme of sample (mg TE/g of dry weight).

##### ABTS (2,2′-Azinobis(3-Ethyl-Benzothiazoline-6-Sulfonic Acid))

The radical scavenging activity of the prepared BSG extracts was measured using an ABTS^+^ radical cation. The mixed solution of the extract and the ABTS working solution in a 96-well plate was incubated (30 min, at room temperature), and the absorbance of the reacted mixture and the blank were read at 734 nm using a microplate smartReader™ 96 (Accuris Instruments, Benchmark Scientific Inc., Edison, NJ, USA). The percentage inhibition of the ABTS radical was calculated using the recorded absorbances.

##### Ferric Ion-Reducing Antioxidant Power

The FRAP assay followed the procedure reported by Xiao et al. [25]. The required solutions, which included an acetate buffer (300 mM, pH 3.6), diluted HCl (40 mM), 2,4,6-Tripyridyl-S-triazine (TPTZ, 10 mM), and ferric chloride hexahydrate (FeCl_3_⋅6H_2_O, 20 mM), were freshly prepared for the assay. Next, 240 µL of the FRAP working solution (at 37 °C) was pipetted into each plate well, followed by 10 µL of the standard (Trolox solution, 0 to 1 mM), 10 µL of the methanolic extracts to another series of wells, and the blank control was 75% ethanol [26]. The plate was incubated at 37 °C for 30 min in an incubator (Model 222/227, Scientific Manufacturing CC, Cape Town, South Africa), and the derived reaction mixture absorbance was read at 593 nm on a microplate smartReader™ 96 (Accuris Instruments, Benchmark Scientific Inc., Edison, NJ, USA). The FRAP of the extracts was further presented as a millimolar of Trolox equivalent per gramme (mM TE/g).

### 2.7. Thermal Properties Using Differential Scanning Calorimeter (DSC)

The thermal properties of the samples were assessed using a DSC system equipped with STARe software (CH-8606, Mettler Toledo, Greifensee, Switzerland), following a modified version of the method by [27]. Approximately 10 mg of the sample was placed in an aluminium pan (Al crimp Pan C.201-52943) using an empty pan as reference, in an atmosphere of nitrogen gas (99.99% purity), with the heating temperature ranging between 25 and 500 °C at a scan rate of 10 °C/m, and held for 1 min at 500 °C. The melting transition characteristics were determined from the endotherms.

### 2.8. Statistical Analysis

The statistical analysis was performed using SPSS V. 22 (SPSS Inc., Chicago, IL, USA). All analyses were replicated and data are presented as mean ± standard deviation. One-way analysis of variance (ANOVA) and Duncan’s multiple tests were used to evaluate the data. Means were compared using Tukey’s multiple range test at the *p* ≤ 0.05 significance level of probability, and values were represented as the mean of standard deviation.

## 3. Results and Discussion

### 3.1. Colour Analysis of BSG Powder

Table 1 shows the colour parameters of BSG flour. The colour of dried flour is important as it determines the quality and sensory attractiveness of the product. Colour is one of the most important parameters consumers use to evaluate food [28]. The pretreatment of BSG significantly affected (*p* < 0.05) the lightness (L* value) and redness (a* value) of the samples. The ultrasonication and fermentation (24 h) of BSG slightly increased L* values, i.e., the samples became lighter, which is especially pronounced in the ultrasonicated sample (15 min). Furthermore, some differences in yellowness (b* value) were not observed for the ultrasonicated samples. A reduction was observed in the redness value after ultrasonication, which decreased further with an increase in ultrasonication time. The fermentation of BSG significantly affected its yellowness, which might be connected to particular metabolites (such as ammonia, amines, and phenolic compounds) produced by microbial actions. Generally, fermentation often leads to a reduction in the Maillard reaction. Researchers have reported reduction in brown colour of BSG-added bread as a result of free sugar reduction by microorganisms during fermentation [29]. Olvera-Ortiz et al. [30] also reported a decrease in the browning index in colour of fermented BSG, with increased lightness thereby improving the colour of the BSG. A combination of applied treatments improves the techno-functional properties of BSG and broadens the diversity of food products.

### 3.2. Physicochemical Properties (pH, Total Titratable Acidity, and Soluble Solids) of Native BSG and Pretreated BSG Flours

Table 2 shows the effect of pretreatment on the physicochemical properties of native BSG and pretreated BSG flours. The value of pH and TTA ranged between 3.97 and 4.31 and 1.50–2.40 mg/g, respectively. The pH and TTA of pretreated BSG showed a statistically significant (*p* < 0.05) increase after ultrasonication and fermentation. Fermented BSG had lower pH, while TTA values were higher than native BSG. Mudau and Adebo [31] observed similar trends in the fermentation and ultrasonication of Bambara groundnut flour. This result could be attributed to the breakdown of complex organic molecules by cavitation and microorganisms during ultrasonication and fermentation, respectively, which led to the build-up of organic acids such as lactic and citric acids, ultimately leading to the acidity of the pretreated BSG. Generally, *Lactobacillus* spp. has been reported to raise the acidity to high levels in the fermentation process.

The general increase in TSS of the pretreated BSG could be linked to the accumulation of sugar due to the increased activities of enzymes during fermentation. However, Eliopoulos et al. [32] reported a decrease in the concentration value (10.34–7.66%, Day 0–Day 12) of TSS in the solid fermentation of BSG. This is due to microbes and the variety of the BSG used.

### 3.3. Proximate Composition of BSG Flour

The BSG flour showed a moisture content of 3.59–7.11% (Table 3), with the 30 min ultrasonicated BSG flour displaying the highest value (7.11%) and the native BSG sample a value of 6.89%. Moisture content (MC) decreased with ultrasonication and fermentation pretreatments, except for the 30 min ultrasonicated sample. The MC of the pretreated BSG flour sample was lower than that of native flours. A similar trend was observed in the ultrasonicated *M. oleifera* Lam. leaf powder by Mudau and Adebo [33] which might not be unconnected to its low dry matter. The importance of MC in food ingredients cannot be over-emphasised as it determines the shelf life of the food product. The fibre content of BSG ranged from 18.50 to 28.12%. The fibre content in the fermented and ultrasonicated samples was significantly higher than in the untreated BSG sample. A comparable rise in the fibre content of various plant foods following high-intensity ultrasound treatment was likewise reported by Kalla-Bertholdt [34]. This development might be due to cavitation and microbial action on the selective fibre fraction during ultrasonication and semi-solid fermentation, respectively [35]. The protein and ash content value ranged from 24.46 to 24.51% and 2.99 to 5.83%, respectively. Generally, there was no significant difference in the protein value of the pretreated samples compared with the untreated samples, while there was a significant increase in the ash value (5.83%) in the sample fermented with CHN-22 (24 h). The increase in ash content might be traceable to the activities of microorganisms on the BSG hemicellulose during fermentation. BSG has been identified as an excellent source of both protein and ash. The protein and ash contents of BSG were similar to those of previous reports [36].

Microbe-mediated processing of BSG has been reported to enhance its nutritional content. Fermentation by certain microbes of BSG degrades lignocellulosic materials leading to a significant increase in the nutritional content of BSG by fermentation [37]. BSG fermentation using strains of fungi with a solid-state method has successfully increased the levels of amino nutrients and reduced the levels of carbohydrates, fats, and dietary fibre [38].

### 3.4. Total Phenolic Content (TPC)

The TPC of the pretreated BSG (0.37–1.26 mg GAE/g) showed a statistically significantly (*p* < 0.05) lower value when compared with the control (1.42 mg GAE/g) (Table 3). Ji et al. [39] reported a similar decrease in phenolic compounds of ultrasonicated coffee leaves. The reduction in total phenolic compound might be attributed to localised heat which might cause the thermal degradation of phenolic compounds as well as free radical formation which may oxidise phenolic compounds. The fermentation with *Lactococcus lactis* subsp. *Lactis*, CHN-22, CHR Hansen, and *Lactobacillus debrueckii* subsp. *bulgaricus*, (YC-XII) could not release the bound phenolic compounds from the BSG flour. This result might be attributed to the barley cultivar and presence of the hull which might have affected accessibility of bound phenolics [40]. Furthermore, limited enzymatic activity of the specific LAB strains used and the fermentation conditions may not have been optimal for effective and maximal release of specific phenolic compounds.

### 3.5. Total Flavonoid Content (TFC)

The TFC, expressed as mg QE/g, of the pretreated BSG (0.13–0.16 mg QE/g) showed a statistically significant (*p* < 0.05) higher value compared with the control (0.13 mg QE/g) (Table 4). BSG phenolics are mostly present in a bound state, and the hydrolytic activities of the fermenting microbial enzymes could lead to a more significant structural degradation of the BSG cell walls, thereby influencing the transformation of insoluble-bound polyphenolics to their free form [40]. Zhang et al. [41] reported a similar increase in total flavonoid content of ultrasonicated germinated black highland barley. This outcome could be attributed to factors such as barley type and the inclusion or exclusion of hull. The variation in response between TPC and TFC suggests differential effects of the pretreatments on various phenolic subclasses. Phenolic and flavonoid content could provide an insight into the potential health-promoting benefits of incorporating BSG into food products. Although better chromatographic instrumental methods will be required to provide a better overview and adequately identify and quantify the phenolic compounds in the samples.

### 3.6. Antioxidant Activity of Native BSG and Pretreated BSG

Table 5 shows the results of the percentage of the inhibition of DPPH of native BSG and different samples of pretreated BSG. The highest DPPH inhibition value was 65%, and the lowest was 15% for native and fermented (CHN-22, 48 h), respectively. The ABTS activity for BSG flour, as shown in Table 5, was 23.50% inhibition (native sample), while the pretreated samples ranged from 20.29 to 37.29% inhibition. The highest ABTS value was recorded at 15 min of BSG ultrasonication. The enhancement in value might be related to the crack of the BSG cell wall as a result of sonication at a relatively lower duration (15 min). Reports have shown that ultrasonication at higher temperatures does not cause any further damage to the cell wall except fibrillation [42]. The extract from BSG had a FRAP value of 2.10 mg TE/g, and the pretreated samples ranged from 1.67 to 4.35 mgTE/g and showed a significantly higher value (*p* < 0.05) compared to the control. The reducing power (FRAP) assay is frequently utilised to measure phenolic antioxidant activity.

### 3.7. Thermal Properties

**Figure 1:**
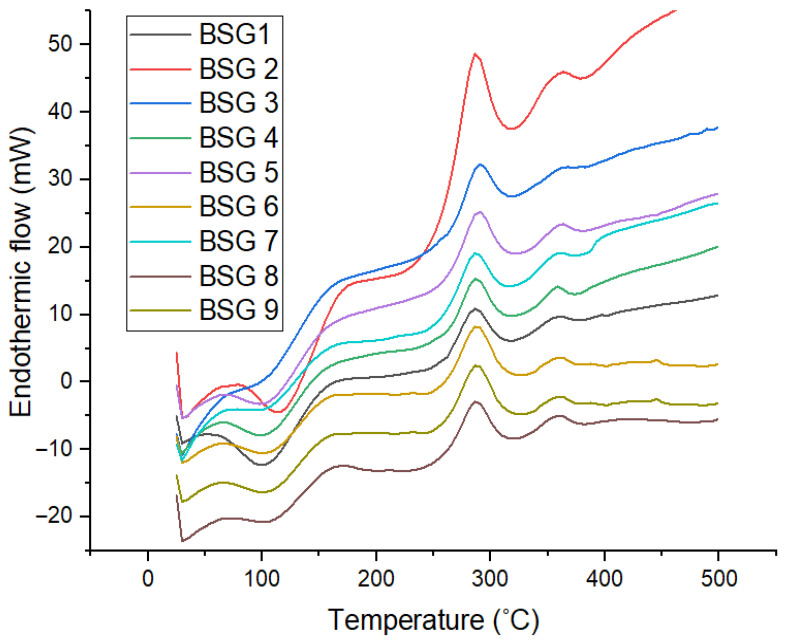
DSC thermogram of native and pretreated BSGs. BSG1—control; BSG2—ultrasonication (15 min); BSG3—ultrasonication (30 min); BSG4—fermented with YC-X11 (24 h); BSG5—fermented with YC-X11 (48 h); BSG6—fermented with YC-X11 (72 h); BSG7—fermented with CHN-22 (24 h); BSG8—fermented with CHN-22 (48 h); and BSG9—fermented with CHN-22 (72 h).

Thermal properties of the native (control) and treated samples were investigated using DSC. Figure 1 shows the DSC thermograms for samples obtained at different temperatures ranging from 10 to 500 °C. Both native (control) and pretreated BSG displayed a prominent endothermic peak or shoulder, peaking broadly around 80–120 °C. This suggests the evaporation of free and bound water, while the intensity of the peak intensity indicates differences in moisture content and water-binding capacity as influenced by ultrasonication and fermentation. While the water-binding capacity was not investigated in this study, such differences in Figure 1 are also reflected in the moisture content of the samples (Table 3). With an increase in temperature, upward progressing peaks were observed between 150 and 300 °C which generally indicates the sample transitioning from solid to liquid, while subsequent broad peaks > 300 °C correspond to the thermal decomposition of structural carbohydrates (e.g., hemicellulose) and lignin breakdown. It could be postulated that ultrasonication disrupted the structure of BSG via cavitation, leading to a re-organisation of the polysaccharide structure [43,44,45], contributing to heightened endothermic flow. Through microbial activity, fermentation on the other hand biochemically hydrolysed various components of BSG [46], progressively reducing peak intensities.

## 4. Conclusions

Research focus is shifting towards enhancing the health-promoting properties of brewers’ spent grains as functional food ingredients. This study analysed and characterised the ultrasound- and fermentation-assisted pretreatment of BSG in an aqueous medium. By demonstrating that simple, scalable pretreatment strategies can significantly enhance the properties of BSG, this study provides a foundation for the development of BSG-enriched food products. The choice between these pretreatments should however be guided by the specific functional requirements of the intended application, with both approaches offering viable pathways for BSG valorisation in the food industry. Additional research using metabolomic profiling techniques is however required to gain a better understanding about how these pretreatments affect the composition and quantities of phytochemicals and other important metabolites. Furthermore, it is important to also investigate how the pretreatments used in this study affect the inherent functional (oil and water holding) as well as rheological properties of BSG. The findings in this study are a steppingstone towards developing food products composited with ultrasonicated or fermented BSG which could contribute towards addressing global challenges related to food waste, dietary fibre deficiency, and the negative effects associated with oxidative stress.

## Figures and Tables

**Table 1:** L*, a*, and b* values of pretreated BSG.

BSG	L*	a*	b*
1	57.43 ± 0.85 ^b^	3.47 ± 0.30 ^ab^	9.88 ± 0.29 ^bcd^
2	57.10 ± 0.21 ^bc^	3.26 ± 0.02 ^ab^	9.61 ± 0.02 ^d^
3	57.84 ± 0.61 ^ab^	3.18 ± 0.07 ^c^	9.71 ± 0.08 ^cd^
4	57.75 ± 0.52 ^ab^	3.63 ± 0.04 ^a^	10.55 ± 0.29 ^a^
5	57.45 ± 0.88 ^b^	3.59 ± 0.06 ^ab^	10.48 ± 0.27 ^ab^
6	57.08 ± 0.59 ^bc^	3.45 ± 0.16 ^ab^	10.41 ± 0.27 ^abc^
7	56.92 ± 0.08 ^c^	3.27 ± 0.07 ^bc^	10.16 ± 0.15 ^ab^
8	56.33 ± 0.45 ^b^	3.58 ± 0.09 ^ab^	9.91 ± 0.92 ^bcd^
9	59.09 ± 0.13 ^a^	3.31 ± 0.04 ^bc^	10.70 ± 0.41 ^a^

Different letters in superscript indicate significant differences (Tukey’s test, *p* < 0.05). BSG1—Control; BSG2—Ultrasonication (15 min); BSG3—ultrasonication (30 min); BSG4—fermented with YC-X11 (24 h); BSG5—fermented with YC-X11 (48 h); BSG6—fermented with YC-X11 (72 h); BSG7—fermented with CHN-22 (24 h); BSG8—fermented with CHN-22 (48 h); and BSG9—fermented with CHN-22 (72 h).

**Table 2:** Physicochemical properties (pH, total titratable acidity, and soluble solids) of control BSG and pretreated BSG flours.

BSG	pH	TTA (mg/g)	TSS
1	3.97 ± 1.00 ^a^	1.50 ± 0.10 ^de^	1.50 ± 0.10 ^c^
2	4.31 ± 0.03 ^a^	2.00 ± 0.10 ^c^	1.40 ± 0.00 ^c^
3	4.27 ± 0.01 ^a^	2.30 ± 0.10 ^b^	1.40 ± 0.00 ^c^
4	4.27 ± 0.01 ^a^	2.40 ± 0.44 ^ab^	2.73 ± 0.11 ^b^
5	4.29 ± 0.01 ^a^	1.16 ± 0.01 ^c^	2.90 ± 0.00 ^ab^
6	4.25 ± 0.01 ^a^	1.50 ± 0.10 ^b^	3.23 ± 0.12 ^a^
7	4.29 ± 0.00 ^a^	1.70 ± 0.10 ^d^	2.80 ± 0.00 ^abc^
8	4.10 ± 0.56 ^a^	2.40 ± 0.10 ^ab^	1.57 ± 0.01 ^c^
9	4.43 ± 0.04 ^a^	2.70 ± 0.10 ^a^	3.03 ± 0.25 ^ab^

Different letters in superscript indicate significant differences (Tukey’s test, *p* < 0.05). BSG1—control; BSG2—ultrasonication (15 min); BSG3—ultrasonication (30 min); BSG4—fermented with YC-X11 (24 h); BSG5—fermented with YC-X11 (48 h); BSG6—fermented with YC-X11 (72 h); BSG7—fermented with CHN-22 (24 h); BSG8—fermented with CHN-22 (48 h); and BSG9—fermented with CHN-22 (72 h).

**Table 3:** The chemical composition of pretreated BSG flour.

BSG	Moisture Content (%)	Fibre (%)	Protein (%)	Ash (%)
1	6.89 ± 0.03 ^ab^	18.50 ± 0.21 ^d^	24.51 ± 0.03 ^a^	3.73 ± 1.16 ^a^
2	4.45 ± 0.03 ^cd^	20.05 ± 1.02 ^cd^	24.34 ± 0.19 ^bc^	2.97 ± 0.03 ^d^
3	7.11 ± 0.03 ^a^	24.86 ± 1.48 ^ab^	24.51 ± 0.25 ^a^	5.39 ± 0.13 ^ab^
4	5.20 ± 0.06 ^c^	22.18 ± 1.69 ^bc^	24.41 ± 0.25 ^abc^	4.94 ± 0.11 ^b^
5	5.52 ± 0.09 ^b^	24.53 ± 1.92 ^b^	23.96 ± 0.14 ^cd^	5.19 ± 0.11 ^b^
6	5.58 ± 0.07 ^b^	21.67 ± 1.48 ^c^	23.86 ± 0.16 ^d^	3.30 ± 0.11 ^cd^
7	3.65 ± 0.04 ^d^	28.12 ± 4.79 ^a^	24.40 ± 0.15 ^abc^	5.83 ± 0.00 ^a^
8	3.59 ± 0.06 ^d^	26.48 ± 14.78 ^ab^	24.46 ± 0.20 ^ab^	3.19 ± 0.05 ^d^
9	5.29 ± 0.80 ^bc^	18.79 ± 2.38 ^d^	24.26 ± 0.01 ^c^	2.99 ± 0.14 ^d^

Different letters in superscript indicate significant differences (Tukey’s test, *p* < 0.05). BSG1—control; BSG2—ultrasonication (15 min); BSG3—ultrasonication (30 min); BSG4—fermented with YC-X11 (24 h); BSG5—fermented with YC-X11 (48 h); BSG6—fermented with YC-X11 (72 h); BSG7—fermented with CHN-22 (24 h); BSG8—fermented with CHN-22 (48 h); and BSG9—fermented with CHN-22 (72 h).

**Table 4:** Total phenolic content (TPC) and total flavonoids content (TFC).

BSG	TPC (mg GAE/g)	TFC (mg QE/g)
1	1.42 ± 2.2 ^a^	0.13 ± 0.0 ^b^
2	0.37 ± 0.05 ^d^	0.14 ± 0.00 ^ab^
3	0.86 ± 0.42 ^c^	0.14 ± 0.0 ^ab^
4	1.30 ± 0.08 ^ab^	0.13 ± 0.01 ^b^
5	1.30 ± 0.22 ^ab^	0.14 ± 0.01 ^ab^
6	1.12 ± 0.08 ^bc^	0.13 ± 0.01 ^b^
7	0.75 ± 0.08 ^d^	0.16 ± 0.01 ^a^
8	1.26 ± 0.01 ^b^	0.14 ± 0.01 ^ab^
9	0.79 ± 0.63 ^cd^	0.13 ± 0.01 ^b^

Different letters in superscript indicate significant differences (Tukey’s test, *p* < 0.05) BSG1—control; BSG2—ultrasonication (15 min); BSG3—ultrasonication (30 min); BSG4—fermented with YC-X11 (24 h); BSG5—fermented with YC-X11 (48 h); BSG6—fermented with YC-X11 (72 h); BSG7—fermented with CHN-22 (24 h); BSG8—fermented with CHN-22 (48 h); and BSG9—fermented with CHN-22 (72 h).

**Table 5:** Antioxidant activity of native and pretreated BSG.

BSG	FRAP (mgTE/g)	DPPH (% Inhibition)	ABTS (% Inhibition)
1	2.10 ± 0.35 ^c^	65.87 ± 3.56 ^ab^	23.50 ± 0.90 ^bc^
2	4.35 ± 0.80 ^a^	66.76 ± 1.19 ^a^	37.29 ± 10.09 ^a^
3	3.44 ± 0.10 ^ab^	64.67 ± 3.65 ^bc^	20.29 ± 1.43 ^cd^
4	2.25 ± 0.42 ^bc^	57.63 ± 71.44 ^b^	27.60 ± 0.61 ^abc^
5	2.29 ± 0.25 ^bc^	63.92 ± 1.86 ^c^	24.48 ± 3.88 ^bc^
6	1.67 ± 0.12 ^d^	57.18 ± 30 ^d^	28.44 ± 10.46 ^ab^
7	2.01 ± 0.07 ^cd^	57.93 ± 1.58 ^cd^	22.25 ± 4.75 ^c^
8	2.43 ± 0.56 ^b^	61.23 ± 0.01 ^a^	22.25 ± 4.75 ^c^
9	2.14 ± 0.36 ^c^	65.27 ± 1.82 ^b^	30.58 ± 6.42 ^ab^

Different letters in superscript indicate significant differences (Tukey’s test, *p* < 0.05). BSG1—control; BSG2—ultrasonication (15 min); BSG3—ultrasonication (30 min); BSG4—fermented with YC-X11 (24 h); BSG5—fermented with YC-X11 (48 h); BSG6—fermented with YC-X11 (72 h); BSG7—fermented with CHN-22 (24 h); BSG8—fermented with CHN-22 (48 h); and BSG9—fermented with CHN-22 (72 h).

## Data Availability

The data presented in this study are available on request from the corresponding authors.
